# Continuous and Stable Printing Method of Planar Microstructure Based on Meniscus-Confined Electrodeposition

**DOI:** 10.3390/ma17184650

**Published:** 2024-09-23

**Authors:** Yawen Yang, Hanchi Wan, Qiang Xing, Xiaoping Zhang, Haili Xu

**Affiliations:** School of Mechanical Engineering, Nantong University, Nantong 226019, China; 2210310009@stmail.ntu.edu.cn (Y.Y.); vans@stmail.ntu.edu.cn (H.W.); meexq@ntu.edu.cn (Q.X.); zhang.xp@ntu.edu.cn (X.Z.)

**Keywords:** meniscus-confined electrodeposition, plane adaptability, finite element method, additive manufacturing

## Abstract

The meniscus-confined electrodeposition (MCED) technique offers advantages such as low cost and wide applicability, making it a promising method in the field of micro/nanofabrication. However, unstable meniscal morphology and poor deposition quality during planar deposition in MCED necessitate the development of improved methods. Therefore, a planar adaptive micro-tuning deposition method (PAMTDM), which utilizes the positioning technology of scanning electrochemical cell microscopy (SECCM) and employs a singular value decomposition (SVD) planar fitting method to determine the flatness of the deposition plane, is proposed. An adaptive micro-tuning motion mode was proposed by analyzing the variation patterns of the meniscus. Moreover, a combination of multi-physics finite element simulations and orthogonal experimental methods was introduced to determine the optimal motion parameters. The experimental results demonstrate that the PAMTDM effectively addresses the issues encountered during planar growth. Compared to the point-by-point deposition method, the PAMTDM achieves a threefold increase in deposition speed for continuous deposition of 105-μm-long line segments in two-dimensional planes, with a deposition current error of less than 0.2 nA. In conclusion, the proposed method provides significant insights into the broad future applications of MCED.

## 1. Introduction

Electrochemical deposition, a typical additive manufacturing technique used to construct three-dimensional structures using layer-by-layer stacking materials [1], is characterized by a wide range of applicable materials and advantages such as fewer deposition condition restrictions and high controllability. Electrochemical deposition methods at the micro/nanoscale include Lithographie, Galvanoformung, Abformung (LIGA), localized electrodeposition, and jet electrochemical deposition [2]. However, these methods are often associated with high processing costs and stringent environmental requirements, limiting their practical application. As an alternative, meniscus-confined electrodeposition (MCED), an electrochemical deposition method based on glass microprobes that enables high-resolution and high-precision deposition [3], offers flexibility in application formats, low cost, and suitability for various structural scales [4,5,6].

Hu et al. introduced MCED technology in 2010, demonstrating its potential in the microelectronics industry by “writing” three-dimensional structures of pure copper and platinum. They successfully fabricated high-density, high-quality interconnected structures, highlighting the application prospects of MCED technology [7]. Seol et al. utilized MCED technology to precisely control voltage amplitudes and duty cycles during electrodeposition, guiding the manufacturing of micro/nanoscale pillar-shaped metal structures with solid and hollow configurations. This study demonstrated MCED’s capability to accurately control the internal morphology of metal structures at the microscopic scale, enabling diverse 3D metal microarchitectures [8]. Zhuang et al. conducted a comparative analysis of MCED and scanning electrochemical cell microscopy (SECCM), focusing on system configurations and operational principles. They proposed an alternating current scanning mode for SECCM, facilitating the in situ morphological imaging of microstructures deposited via MCED [9,10]. These studies collectively underscore MCED’s versatility and precision of MCED in microstructure fabrication, providing the foundation for its continued advancement and broader applications in additive manufacturing and microelectronics.

Previous electrochemical deposition studies based on MCED technology have primarily focused on vertical growth structures, which are relatively simple; moreover, these electrodeposition methods cannot deposit complex three-dimensional structures [11,12,13], with planar printing with layer-by-layer stacking required. However, research on planar deposition using MCED technology remains limited. In 2018, Behroozfar et al. utilized localized square-wave electrodeposition technology to adjust printing parameters and achieve planar writing of nanotwinned copper, thereby realizing layered printing of structures [14]. In the same year, Yu et al. proposed a dynamic scanning mode for the MCED technology, which precisely positioned and printed copper lines with uniform width, smooth surfaces, and excellent electrical conductivity [15]. Subsequently, the YU team further developed the MCED technology to control the formation of three-dimensional copper wires based on the quasi-static stability of the meniscus, resulting in straight and uniform copper microwires [16]. However, in practical deposition processes, uncertainty in the flatness of the deposition plane often leads to meniscus instability, causing various problems. For instance, if the distance between the glass pipette tip and the deposition plane is too small, the tip can be blocked by deposits or collide with the substrate, causing it to break. Conversely, if the distance is too large, the meniscus can stretch excessively or fail to form, interrupting deposition. Meniscus instability can lead to poor deposition quality and rough microstructure surfaces, preventing stable and continuous planar growth deposition. Therefore, addressing these challenges is crucial for advancing the application of the MCED technology to the planar deposition of complex three-dimensional structures.

Therefore, this study leverages the scanning positioning capabilities of SECCM to derive the planar flatness equation of a deposition substrate using singular value decomposition (SVD). Additionally, based on the movement characteristics of SECCM and the stretching and contraction patterns of the meniscus, we designed an adaptive micro-tuning motion mode to effectively address the issues arising during continuous planar writing. Combining a multi-physics finite element simulation to analyze height variations during the deposition process with an orthogonal experimental method, we determined the optimal motion parameters to achieve rapid and stable planar manufacturing at the microscale. The planar adaptive micro-tuning deposition method (PAMTDM) results in microstructures with uniform surfaces, high deposition speeds, and excellent deposition stability, independent of the level of the deposition plane, thus highlighting its considerable potential for widespread application in manufacturing micro-precision structures.

## 2. Principles and Methods

### 2.1. Principle of Meniscus-Confined Electrodeposition

**Figure 1:**
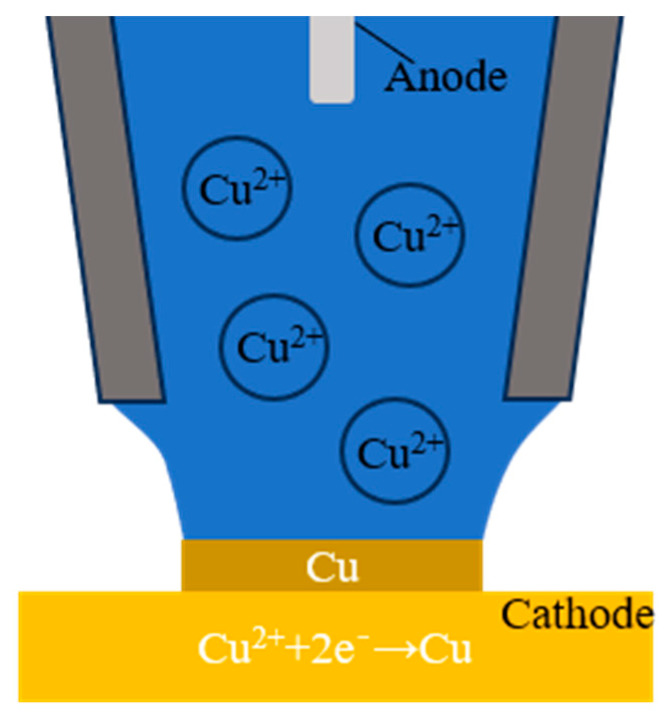
Principles of meniscus-confined electrodeposition (MCED).

MCED primarily utilizes a glass micropipette (Borosilicate capillary tube with an outer diameter of 1.5 mm and a length of 10 cm, produced by Sutter Company, model BF100-58-10). with a 5-μm opening at the tip, filled with a solution containing metal ions—0.1 M CuSO_4_ solution was used in this study—as the main experimental setup, as illustrated in Figure 1. The micropipette was filled with a CuSO_4_ solution, with an inert electrode inserted to serve as the anode, while the conductive substrate acted as the cathode for electrochemical deposition. As the glass micropipette approached the conductive substrate, a meniscus formed between the two. Upon applying an appropriate voltage, the copper ions in the solution migrate to the surface of the conductive substrate under the combined effects of convection, diffusion, and electromigration, where they undergo reduction reactions. This process led to the deposition of elemental Cu on the conductive substrate.

### 2.2. Planar Adaptive Micro-Tuning Deposition Method (PAMTDM)

**Figure 2:**
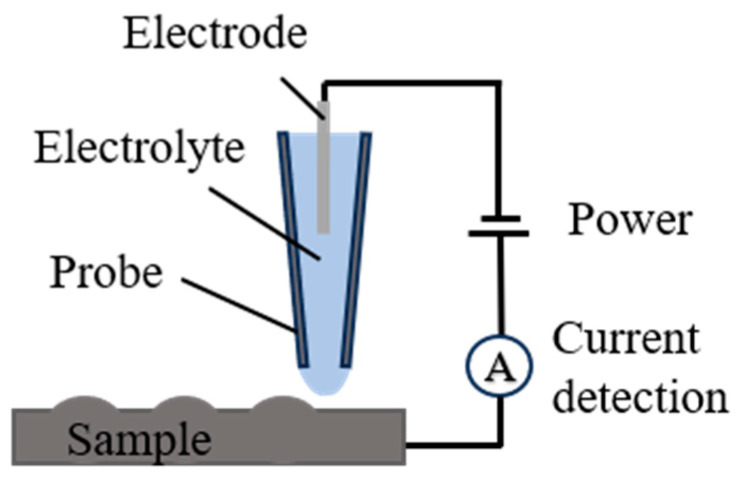
SECCM schematic diagram.

During the two-dimensional planar deposition of the microstructures, the meniscus is small and unstable and thus requires a highly flat deposition plane. In practical deposition processes, maintaining the absolute level of the deposition plane is challenging, often resulting in the glass micropipette tip breaking or the deposition meniscus disappearing, leading to deposition interruption. SECCM, first proposed by Unwin et al. and shown in Figure 2, is a technique that can measure the surface morphology of a sample by generating a tiny droplet (electrochemical cell) at the probe tip when in contact with the sample under a bias voltage. SECCM can acquire positional information on the deposition plane [17,18,19,20]. Therefore, based on the similarity in the physical configurations of the SECCM and MCED techniques, this study proposes a PAMTDM based on SVD fitting.

#### 2.2.1. SVD Plane Fitting

First, the work area level was detected, following which the probe was moved to the four vertices of the deposition plane, where an alternating voltage was applied. Contact between the glass micropipette and the deposition plane resulted in a tiny droplet, generating current feedback. This feedback provides the height coordinates of these four points, thereby obtaining the spatial coordinates of the four points that constitute the deposition plane. SVD is then used to perform planar fitting.

Assuming that the centroid coordinates of the four points are $\left(\bar{x},\bar{y},\bar{z}\right)$, the coordinates of all the points are subtracted from the centroid coordinates to obtain the centered coordinates. The centered matrix *β* can be written as the following:

(1) $$\beta =\left[\begin{array}{ccc} x_{1}-\bar{x} & y_{1}-\bar{y} & z_{1}-\bar{z}\\ x_{2}-\bar{x} & y_{2}-\bar{y} & z_{2}-\bar{z}\\ \vdots & \vdots & \vdots \\ x_{4}-\bar{x} & y_{4}-\bar{y} & z_{4}-\bar{z} \end{array}\right]$$

This section is divided into several subsections, providing a concise and precise description of the experimental results, their interpretation, and experimental conclusions.

Next, performing SVD on the centered matrix *β* yields the following:

(2) $$\beta =UDV^{T}$$

where *D* is a diagonal matrix and *U* and *V* are orthogonal matrices.

In an ideal scenario, all spatial points lie within the fitted plane. However, owing to the micron scale of the substrate and meniscus during deposition and the inherent measurement errors in SECCM, some points may fall outside the plane, thereby reducing the fitting accuracy. Therefore, minimizing the sum of the projection lengths of the coordinate points onto the normal vector of the fitted plane is necessary, expressed as follows:

(3) $$F=\min\left\|\beta n\right\|$$

where n is the last column of matrix *V*, representing the normal vector of the deposition plane, n=(A,B,C).

The normal vector is normalized to satisfy the following:

(4) $$A_{n}^{2}+B_{n}^{2}+C_{n}^{2}=1$$

Finally, substituting the centroid into the equation yields the plane equation:

(5) $$A_{n}x+B_{n}y+C_{n}z=H$$

where *x*, *y*, and *z* are the coordinates of the measurement points, $\left[A_{n}\;B_{n}\;C_{n}\right]$ is the normalized normal vector, and *H* is the constant term of the plane equation.

**Figure 3:**
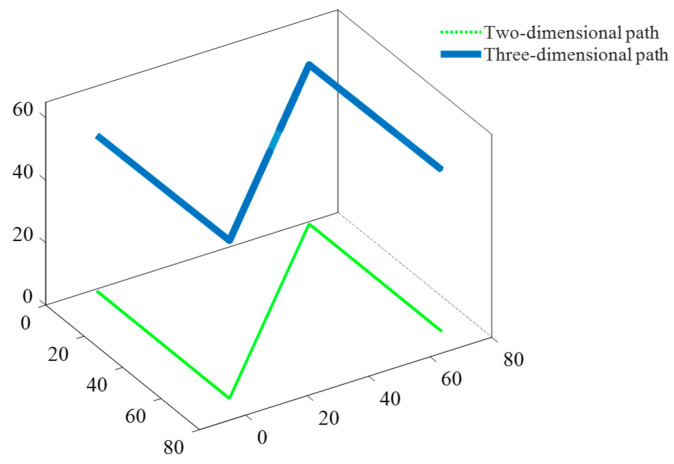
Illustration of three-dimensional deposition paths parallel to the deposition plane.

The eigenvector corresponding to the smallest singular value was proportional to the coefficient vector of the fitting plane. Before the deposition, the reference point coordinates were obtained using a probe and substituted into the equation, resulting in a plane equation based on the deposition plane. By applying an algorithm to extract two-dimensional deposition path points from the scanned deposition paths and substituting them into the plane equation, a three-dimensional deposition path parallel to the deposition plane was obtained, as shown in Figure 3. This process effectively resolves the movement conflict between the probe and the deposition plane.

#### 2.2.2. Motion Method

**Figure 4:**
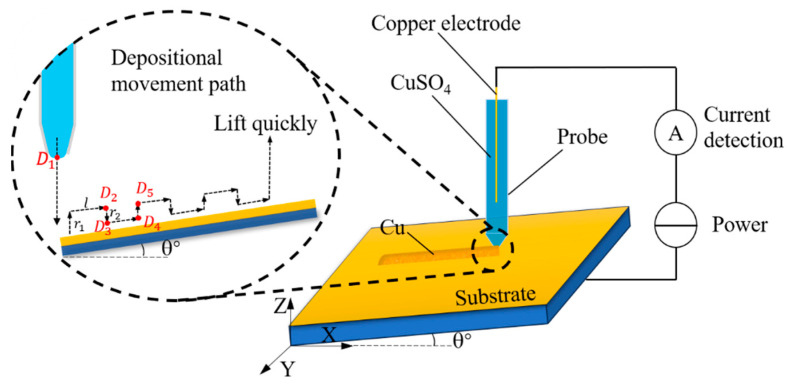
Deposition motion mode.

A fine-tuned deposition motion method was proposed to achieve continuous planar adaptive microstructure deposition based on characteristic changes in the meniscus during deposition at the probe tip. This method uses the physical configuration of MCED combined with the principles of SECCM, with the continuity of the meniscus maintained through a slight jitter-based drag deposition. A schematic of this motion is shown in Figure 4.

After plane fitting, the three-dimensional coordinates of each deposition path point were obtained. If the deposition is performed directly using the jumping scan method of SECCM, it may lead to uneven deposition and poor surface quality. Therefore, an adaptive fine-tuned deposition motion method is proposed using the following workflow:

1. Preparation: Move the probe above the deposition point $D_{1}$ with a distance from the deposition plane not exceeding 80 μm (*Z*-axis range: 0–80 μm). Using the SECCM positioning principle, obtain the Z-coordinate $z_{1}$ of $D_{1}\;$ and set this point as the reference point.
2. Initial Deposition: If deposition proceeds along the *x*-axis, lower the probe from the reference point by $z_{1}$ and observe whether a meniscus forms. If a deposition meniscus forms, time $t_{d}$ is paused to perform the deposition.
3. Upward Lift and Lateral Movement: Lift the probe by $r_{1}$, maintain the meniscus, and move by a distance of *l*. The new position is $D_{2}\left(x_{1}+l,\;y_{1},\;{z_{1}+r}_{1}\right)$.
4. Meniscus Stability Adjustment: To ensure continuous liquid flow at the probe tip and prevent tip clogging due to a small probe opening, lower the glass microprobe by a distance $r_{2}$ ($r_{2}<r_{1}$ to maintain a safe distance from the substrate and avoid collision). The probe tip coordinates at this point are $D_{3}\left(x_{1}+l,\;y_{1},z_{1}+r_{2}\right)$, which stabilize the meniscus through slight pressure.
5. Further Deposition: Move a distance *l* for deposition, reaching point $D_{4}\left(x_{1}+2l,\;y_{1},\;{z_{1}+r}_{2}\right)$.
6. Repetition: Lift the probe to point $D_{5}\left(x_{1}+2l,\;y_{1},\;{z_{1}+r}_{1}\right)$, continue moving the distance *l*, and repeat the above steps.
7. Termination: Upon reaching the endpoint, hold the probe in place for $t_{d}$ to complete the deposition. Finally, lift the probe quickly to complete the deposition process.

**Figure 5:**
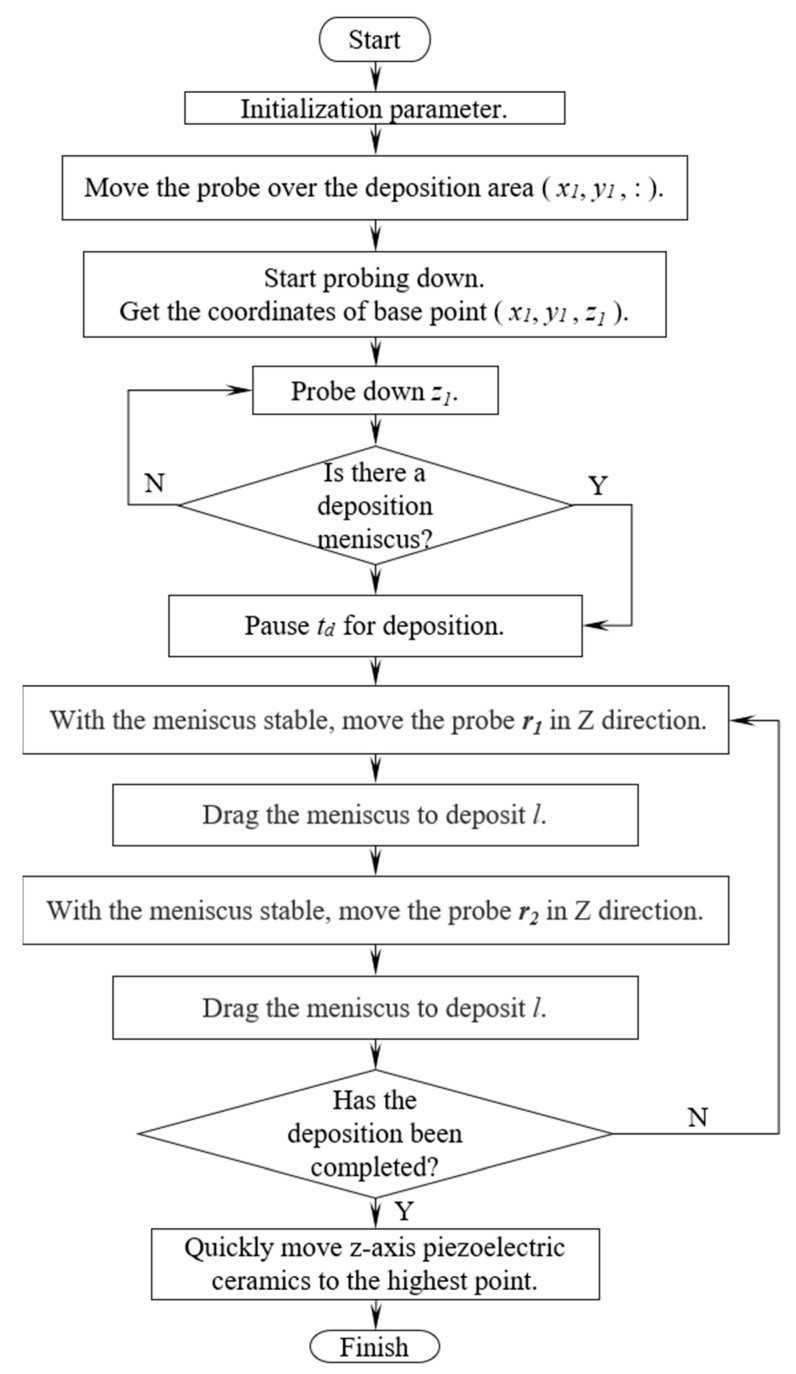
Flowchart of deposition movement.

The motion flow diagram is illustrated in Figure 5.

The equation for the deposition plane was obtained using an SVD plane-fitting algorithm. By combining the extracted two-dimensional deposition path with the plane equation for the deposition plane, a three-dimensional deposition path parallel to the deposition plane is synthesized, thus effectively avoiding issues caused by changes in the distance between the probe tip and conductive substrate during movement. This mode allows for a reasonable arrangement of planar motion trajectories, mitigating phenomena such as incomplete deposition, material accumulation, and uneven deposition lines encountered during direct planar deposition.

## 3. Experimental Platform and Model

### 3.1. Experimental Platform

**Figure 6:**
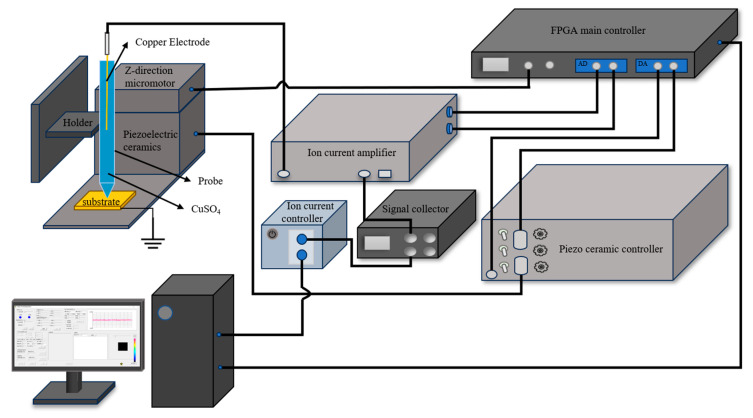
Composition of MCED micro-deposition platform.

The SECCM-based MCED manufacturing platform is presented in Figure 6. The entire system consisted of a glass pipette filled with CuSO_4_ solution, a conductive substrate, a substrate placement platform, a glass pipette clamping platform, an X-Y-Z manual micro-adjustment stage, a *Z*-axis micro-motor coarse adjustment device, an X-Y-Z piezoelectric ceramic fine adjustment device, an ion current controller, a signal acquisition unit, an ion current amplifier, a main controller, and a host computer.

The parameters used in these experiments are listed in Table 1. The 0.1 M copper sulfate solution was prepared using copper sulfate pentahydrate as the solute and ultrapure deionized water. Specifically, a glass capillary with a tip diameter of 5 µm was filled with a 0.1 M CuSO_4_ solution and inserted with an inert electrode as the anode for electrochemical deposition. The ITO conductive glass with dimensions of 10 × 10 × 2 mm was coated with a 200 nm thick gold layer through magnetron sputtering, serving as both the conductive substrate and the cathode in the deposition system [21].

Before each experiment, the surface of the inert electrode needs to be polished to keep it smooth and clean, preventing air bubbles from forming when the electrode is inserted into the glass micro-pipette. Additionally, when filling the glass electrode with CuSO_4_ solution, to avoid air bubbles inside the probe that may affect the experimental results, a second filling is required after the first one to ensure no air enters the tip of the probe.

### 3.2. Multi-Physics Model

When the PAMTDM motion mode is used for planar printing, the optimal vertical movement distance of the glass capillaries must be determined through extensive experimentation. To save time, labor, and resources, this study employed the COMSOL multi-physics 6.1 software to simulate the electrochemical deposition process and analyze the upper and lower limits of the deposition thickness.

**Figure 7:**
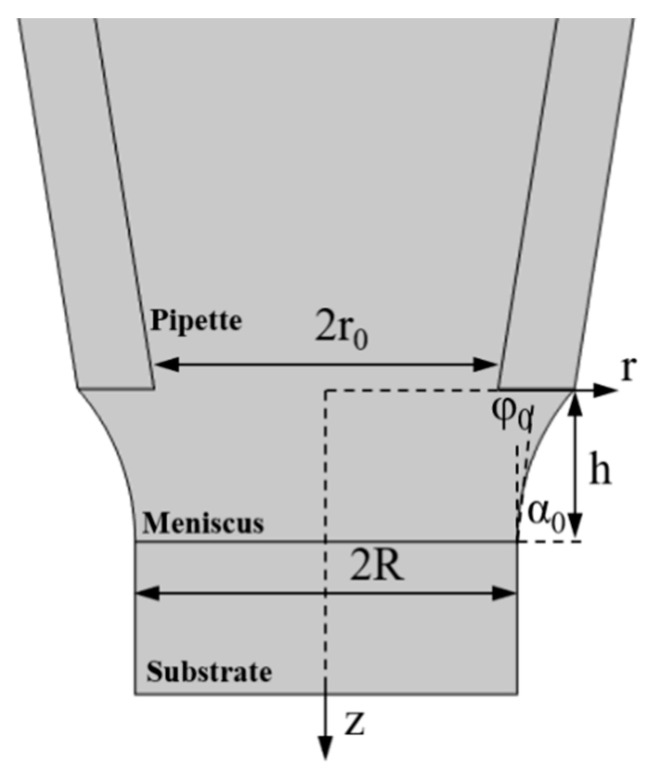
Schematic of the computational geometry.

In this study, finite element analysis was conducted based on the experimental parameters. The geometric model of the MCED is shown in Figure 7, with the meniscus shape described by Equation (6) [22] as follows:

(6) $$z\left(r\right)=Rsin\alpha_{0}\left({cosh}^{-1}\frac{r_{0}}{Rsin\alpha_{0}}-{cosh}^{-1}\frac{r}{Rsin\alpha_{0}}\right)$$

where R and $r_{0}$ represent the radii of the deposited metal and the glass capillary, respectively, while $\varphi_{0}$ is the growth angle, defined as the angle between the tangent of the meniscus and the upper surface of the deposited metal. The relationship is given by $\alpha_{0}=90^{{}^{\circ}}-\varphi_{0}$.

## 4. Optimal Parameter Determination and Experimental Verification

### 4.1. Optimal Parameter Determination

#### 4.1.1. Analysis of Simulation Results

**Figure 8:**
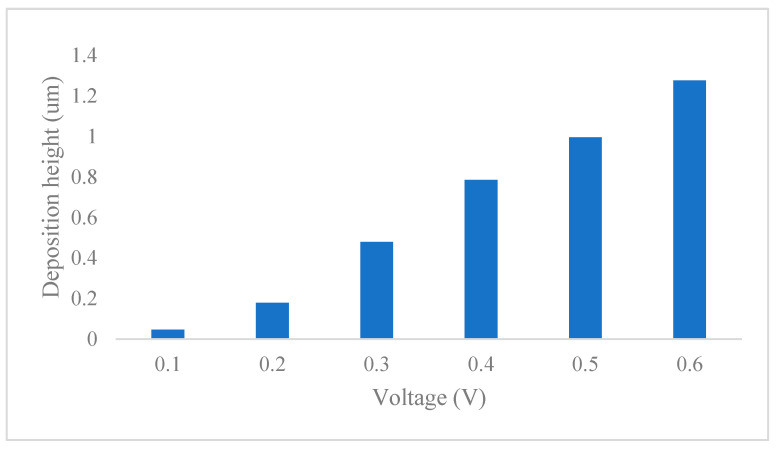
Height of deposition under different voltages.

The externally applied voltage significantly affected the deposition outcome of the MCED. When the deposition voltage is too low, the high impedance at the opening of the glass capillary prevents deposition [23]. Therefore, to determine the appropriate deposition voltage, this study investigated the deposition rate of MCED at voltages ranging from 0.1 to 0.6 V, as shown in Figure 8. As the voltage increases, the deposition height increases for the same deposition time, indicating a higher deposition rate.

**Figure 9:**
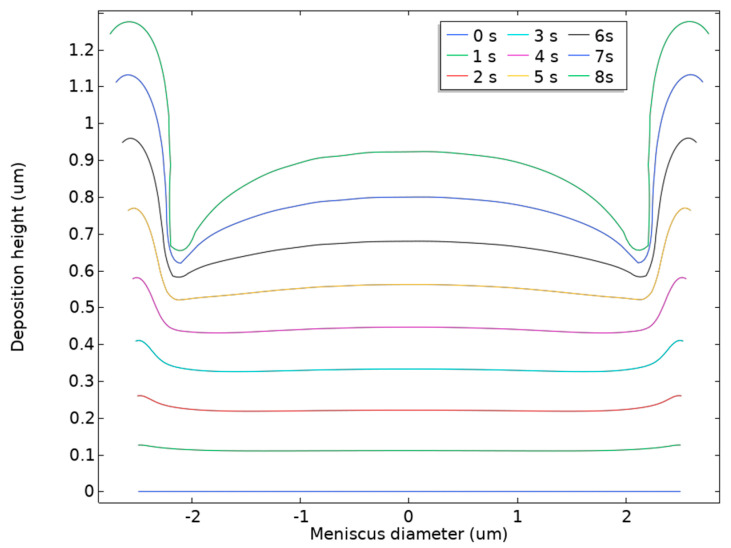
Variation diagram of deposition height. Deposition thickness of the deposits at 0.6 V under different deposition times.

Considering Figure 8, to ensure the quality of the deposition while improving its efficiency, this study selected a deposition voltage of 0.6 V. Based on the characteristics of the PAMTDM motion mode, the optimal adjustment height must also be determined. If the adjustment height is too small, it may cause the glass capillary tip to snag the deposition, which not only disrupts the morphology of the deposition but also risks breaking the tip. To explore the appropriate jumping height, the deposition heights at different durations were observed at a voltage of 0.6 V, as shown in Figure 9.

Owing to the convection and evaporation processes between the liquid bridge and air, the water on the meniscus surface gradually decreases owing to evaporation, resulting in a higher ion concentration at the edges than at the center, subsequently leading to a faster growth rate at the edges of the deposition structure than at the center, as shown by the higher and lower edges in the figure. Metal deposition requires a certain thickness to achieve a uniform and dense deposition structure. As shown in Figure 9, a pause of approximately 1 s was sufficient to initiate the metal deposition. To prevent the probe tip from snagging during deposition and breaking, the downward distance $r_{2}$ of the glass capillary must be greater than 0.1 μm. Continuous deposition was necessary to ensure a uniform surface for the deposited metal. When the glass capillary paused for 5000 ms during its descent, Figure 9 shows that the boundary deposition height reaches nearly 0.8 μm. Therefore, if the upward distance exceeds 0.8 μm, the liquid bridge is likely to break, leading to discontinuous deposition. Thus, $r_{2}$ was selected to be in the range of 0.2–0.8 μm. Additionally, considering electrochemical deposition starts as soon as the meniscus contacts the substrate and a certain thickness of deposition accumulates during the upward movement, the upward distance $r_{1}$ should not be less than $r_{2}$.

#### 4.1.2. Optimal Parameters Based on Experimental Results

During electrochemical deposition at the micrometer scale, the amplitude of each cycle jump significantly affecting the deposition results, with the approximate range of the vertical movement distance of the glass capillary determined through a multi-physics simulation. However, these values require further experimental confirmation. Therefore, an orthogonal experiment was designed to determine the optimal parameters.

This experiment explored the optimal motion parameters for deposition, specifically the vertical movement distances ($r_{1}$ and $r_{2}$) and the step distance l. These parameters were selected as experimental factors, each with four levels. An orthogonal array L_32_ (4^9^) was selected for the experiments, as presented in Table 2. As $r_{1}$ should not be less than $r_{2}$, experiments that did not satisfy this condition were excluded from the orthogonal array. Additionally, each set of parameters was tested thrice, with the results of the orthogonal experiment presented in Table 3. Considering the deposition current during the deposition process is influenced by the stability of the meniscus, balancing deposition quality and efficiency is crucial. Therefore, root mean squared errors (RMSE) of the deposition current and average deposition velocity $\bar{v}$ were chosen as the evaluation metrics.

Based on the data in Table 3, a clear trend can be observed: as the step distance l increases during the deposition process with a glass pipette, and the differences between the upper and lower limits $r_{1}$ and $r_{2}$ decrease, and the average deposition speed for a 70 μm line segment significantly improves. In the experiments, the highest average speed reached 0.486 μm/s, achieved under the conditions where $r_{1}$ and $r_{2}$ are both 0.8 μm and the step distance l is set to 5 μm. However, despite the fastest deposition speed and highest efficiency under these parameters, the root mean square error (RMSE) of the current increased to 0.2062 nA. This error level is relatively high compared to other deposition conditions, indicating instability in the current during high-speed deposition. The RMSE of the current is a crucial indicator for evaluating deposition quality because it reflects current stability, which directly impacts the uniformity and quality of the deposited layer. A high RMSE might indicate significant current fluctuations, which could lead to defects in the deposited layer, such as holes or cracks.

To address this issue, this study proposes a planar adaptive fine-tuning deposition mode aimed at optimizing the stability of the meniscus in the MCED deposition process. When evaluating deposition effects, the RMSE of the current is a key assessment metric, as current stability is vital for maintaining meniscus stability, which directly affects the quality and uniformity of the deposited layer. Analysis of experimental data revealed that Experiment 30 showed the smallest current RMSE among all experiments, at 0.1861 nA. This result indicates that under the conditions of $r_{1}$ = 1.2 μm, $r_{2}$ = 0.8 μm, and step distance l = 3 μm, the meniscus stability is optimal, suggesting that the deposition quality is also the best. With these parameters, current stability was optimized, reducing energy loss and material non-uniformity during the deposition process.

Therefore, through the analysis of different experimental parameter combinations, Experiment 30, with an RMSE of 0.1861, is the lowest among all experiments, demonstrating the best deposition precision and quality. Additionally, Experiment 30’s average deposition speed of 0.4 μm/s, while slightly lower compared to the highest speed of 0.486 μm/s in Experiment 7, still shows high competitiveness in maintaining deposition quality. Particularly, Experiment 30’s superior precision, with its balanced speed and low RMSE, makes it the optimal parameter combination for ensuring both high-quality and efficient deposition.

**Figure 10:**
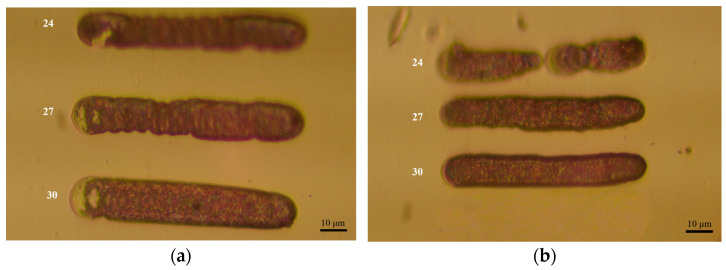
Repeated experiments were conducted using the parameters of experiments 24, 27, and 30. (**a**,**b**) Show the results of two repeated experiments, respectively. In both figures, the experimental outcomes for experiment numbers 24, 27, and 30 are shown from top to bottom.

To further verify the actual printing quality of the optimal deposition parameters, repeated experiments were conducted using the parameters of Experiments 30, 27, and 24, with RMSE values of 0.1861, 0.1965, and 0.2014 nA, respectively. Additionally, the surface morphology of the deposited samples from these experiments was observed using an inverted metallurgical microscope XJL-20, and surface quality was analyzed through image comparison. Morphological comparison results are shown in Figure 10, and the RMSE data comparison results are presented in Table 4.

As shown in Figure 10, the line segments deposited with the parameters of Experiment 30 have a higher success rate, are more continuous, and exhibit a more uniform surface. In contrast, Experiment 24 has a higher failure rate with discontinuous line segments. Although Experiment 27 produces relatively continuous line segments, the surface shows noticeable texture and uneven quality. Analyzing Table 4 further, Experiment 30 has the smallest RMSE, indicating that the current during the deposition process is the most stable under these parameters. Experiment 27 also demonstrates good current stability, whereas Experiment 24 shows poor current stability. Therefore, considering both the actual surface morphology of the deposited results and the current stability during the deposition process, Experiment 30 achieves the best deposition effect while ensuring deposition quality, and also offers a high deposition efficiency.

The optimal deposition parameters are listed in Table 5.

### 4.2. Verification of Deposition Effect of PAMTDM

**Figure 11:**
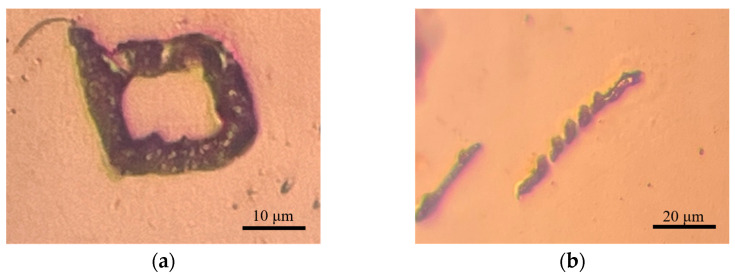

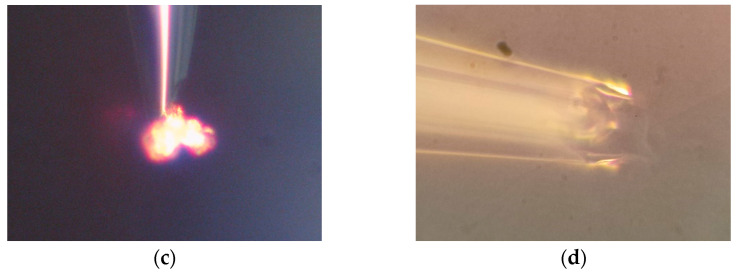
Planar growth deposition experimental results. (**a**,**b**) Are the results of the point-by-point deposition method, (**c**) is a glass pipette blocked by deposits when it is too close to the substrate, and (**d**) is a probe with a broken tip.

Comparative experiments were conducted for planar deposition to validate the feasibility of the PAMTDM motion mode. In the point-by-point deposition experiments based on this planar level, the deposition was uneven, resulting in poor surface roughness and quality. Additionally, instability in the deposition meniscus led to bridge collapse and discontinuous deposition. When the distance between the probe tip and the substrate was too small, the deposition speed increased, causing the copper to block the opening of the glass capillary, resulting in deposition failure. Conversely, when the distance was too large, the risk of probe tip damage increased, as illustrated in Figure 11.

**Figure 12:**
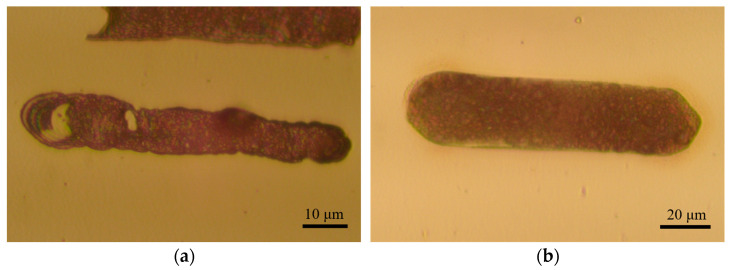
Deposition morphology under different deposition parameters. (**a**) Is the deposition result under non-optimal parameters, and (**b**) is the deposition result under optimal parameters.

**Figure 13:**
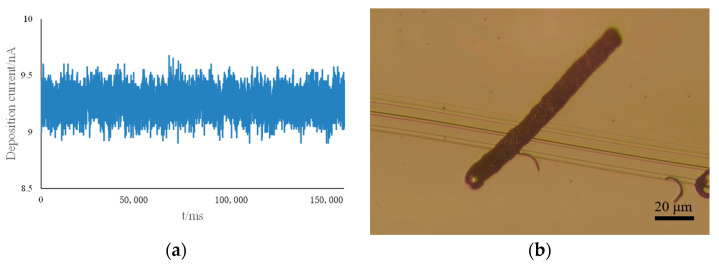

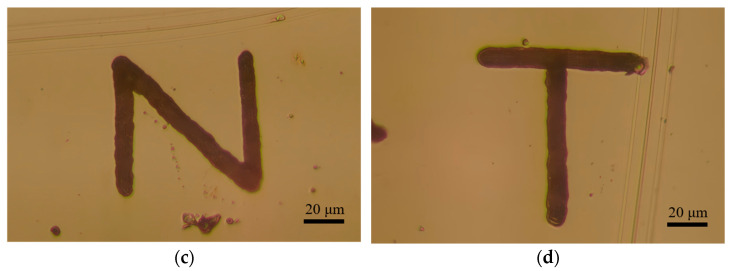
Deposition morphology. (**a**) Is the current record in the deposition process, (**b**) is the experimental result of 105 um long line deposition, and (**c**,**d**) are the deposition effect diagrams of simple letters N and T, respectively.

Subsequent experiments were conducted using the proposed fine-tuned motion model and the optimal parameters obtained from an orthogonal experimental design. The deposition morphology under these parameters, compared with the other parameters, is shown in Figure 12. The deposition quality was more uniform under these optimal parameters. For a deposition line segment of 70 µm in length, the deposition time was approximately 2.5 min, threefold more efficient than the point-by-point deposition method. The current recorded during the deposition process is shown in Figure 13a, indicating stable current variations. Figure 13b shows the result of the long-line deposition experiment, achieving a line length of approximately 105 µm with an aspect ratio of 10.5:1. Figure 13c,d shows the results of the continuous line deposition experiments for simple uppercase letters N and T, demonstrating that the planar adaptive fine-tuning deposition motion mode can achieve the writing of turning lines on a plane. This method can be applied to microcircuit drawing of microelectronic components. However, the deposition results also reflect the issues of blurred and uneven deposition boundaries.

To validate the efficacy of the deposition technique proposed in this study in enhancing deposition rate and improving surface quality, a comparative experiment was designed against the classic point-by-point deposition method. The experimental protocol involved the fabrication of the letter “N” with dimensions of 70 × 70 µm using two distinct deposition techniques. The total time required for deposition using the technique presented in this study was approximately 8 min, achieving nearly a threefold increase in deposition efficiency compared to the point-by-point deposition method.

**Figure 14:**
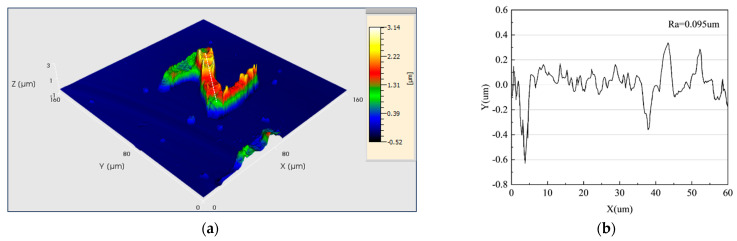

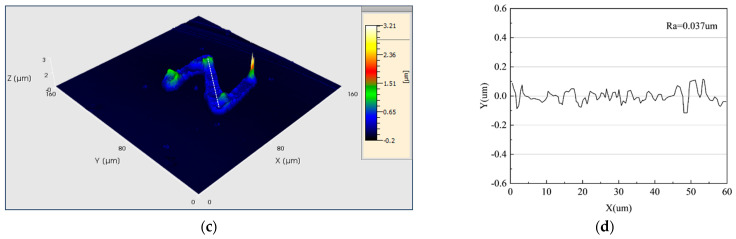
High-resolution confocal microscopy results. (**a**) Three-dimensional topography of the deposition result using the point-by-point deposition method. (**b**) Cross-sectional profile and average roughness at the white dashed line in (**a**). (**c**) Three-dimensional topography of the deposition result using the PAMTDM method. (**d**) Cross-sectional profile and average roughness at the white dashed line in (**c**).

In the assessment of the deposition outcomes, high-resolution confocal microscopy was employed to capture microtopographical images of the surfaces of the samples deposited using both methods and to precisely measure their surface roughness, as illustrated in Figure 14. Figure 14a presents the surface topography of the letter “N” deposited using the point-by-point deposition method, while Figure 14c displays the surface topography of the letter “N” deposited using the deposition technique proposed in this study. Comparative analysis revealed that the samples deposited using the point-by-point method exhibited significant height undulations and lower surface flatness; in contrast, the samples deposited using the PAMTDM method demonstrated a more uniform and smoother surface characteristic. Furthermore, the average surface roughness of the samples deposited using the point-by-point method was 0.095 µm, whereas the average surface roughness of the samples deposited using the PAMTDM method was reduced to 0.037 µm, indicating a significant advantage of the proposed technique in reducing surface roughness. The results indicate that the PAMTDM method outperforms the traditional point-by-point deposition method in both increasing deposition rate and improving surface quality, offering a novel technical approach for the advancement of high-precision deposition technologies.

The experimental results demonstrate that the method proposed in this study enables controlled deposition at the microscale, achieving a processing current RMSE within 0.2 nA. The maximum processing size reached 105 µm, allowing for the drawing of simple planar microstructures. The deposited structures exhibited good surface quality, uniformity, high deposition rate, and stability, thus facilitating the deposition and drawing of multiple lines and planar patterns.

## 5. Conclusions

(1) The positioning function of SECCM was utilized to achieve the adaptive planar micromanufacturing of MCED. Based on the deposition characteristics of the MCED, an adaptive square-wave motion mode suitable for planar deposition growth was designed. Combined with SVD plane fitting, this approach generated a three-dimensional deposition path, effectively mitigating the issues encountered during planar deposition and improving the deposition success rates. Using an orthogonal experimental design, optimal parameters for this motion mode were identified to enhance deposition stability.

(2) Experimental results indicate that the proposed method enables rapid and stable fabrication of planar microstructures, capable of continuously depositing line segments up to 105 μm in length. The deposition speed was three times higher than that of the point-by-point method. While maintaining deposition speed, the proposed method significantly improves the surface quality of the deposited structures, achieving a deposition current RMSE within 0.2 nA, resulting in more uniform structures. Therefore, this method has low deposition costs, a high success rate, and is not limited by the leveling of the deposition plane. It also features a high degree of automation, making it suitable for fabricating various precise metal microstructures and microcomponents. However, due to the limitations of the experimental platform’s motion range, it is currently only capable of printing within an 80 × 80 × 80 μm size, restricting the ability to print larger patterns. Consequently, further research on cross-scale deposition is necessary to broaden its applications in fields such as precision device manufacturing and biomedical sensing.

## Figures and Tables

**Table 1:** Parameters used in the experiment.

Parameter	Value
Copper ion solution	CuSO_4_ (0.1 M)
Glass pipette aperture	5 µm
Rate of travel	1.5 μm/s
Ambient temperature	Room temperature (20 °C)
Environment humidity	40%

**Table 2:** Orthogonal table.

Levels	Factors		
	$r_{1}$	$r_{2}$	*l*
1	0.6	0.2	2
2	0.8	0.4	3
3	1.0	0.6	4
4	1.2	0.8	5

**Table 3:** Results of orthogonal test.

Experimental Serial Number	$r_{1}$/(μm)	$r_{2}$/(μm)	*l*/(μm)	RMSE/(nA)	$\bar{v}$/(μm/s)
1	0.6	0.2	2	0.2286	0.304
2	0.6	0.4	3	0.1976	0.421
3	0.6	0.6	4	0.1923	0.482
4	0.8	0.2	2	0.2196	0.337
5	0.8	0.4	3	0.1872	0.404
6	0.8	0.6	4	0.1936	0.464
7	0.8	0.8	5	0.2062	0.486
8	1.0	0.2	3	0.1971	0.377
9	1.0	0.4	2	0.1876	0.337
10	1.0	0.6	5	0.1987	0.461
11	1.0	0.8	4	0.2033	0.464
12	1.2	0.2	3	0.1896	0.362
13	1.2	0.4	2	0.2017	0.315
14	1.2	0.6	5	0.1992	0.446
15	1.2	0.8	4	0.1929	0.446
16	0.6	0.2	5	0.2004	0.458
17	0.6	0.4	4	0.1918	0.461
18	0.6	0.6	3	0.1953	0.442
19	0.8	0.2	5	0.1960	0.449
20	0.8	0.4	4	0.2007	0.456
21	0.8	0.6	3	0.1908	0.421
22	0.8	0.8	2	0.1884	0.368
23	1.0	0.2	4	0.1968	0.424
24	1.0	0.4	5	0.2014	0.443
25	1.0	0.6	2	0.1877	0.352
26	1.0	0.8	3	0.1863	0.414
27	1.2	0.2	4	0.1965	0.409
28	1.2	0.4	5	0.1989	0.435
29	1.2	0.6	2	0.1901	0.338
30	1.2	0.8	3	0.1861	0.4

**Table 4:** RMSE contrast results.

Experimental Serial Number	RMSE1/(nA)	RMSE2/(nA)
24	0.1944	0.2153
27	0.1596	0.136
30	0.1157	0.1197

**Table 5:** Optimum deposition parameters.

Deposition Parameters	
r_1_/µm	1.2
r_2_/µm	0.8
t_d_/ms	5000
*l*/µm	3

## Data Availability

The original contributions presented in the study are included in the article, further inquiries can be directed to the corresponding author.
